# Kaempherol and Luteolin Decrease Claudin-2 Expression Mediated by Inhibition of STAT3 in Lung Adenocarcinoma A549 Cells

**DOI:** 10.3390/nu9060597

**Published:** 2017-06-13

**Authors:** Hiroyuki Sonoki, Asami Tanimae, Satoshi Endo, Toshiyuki Matsunaga, Takumi Furuta, Kenji Ichihara, Akira Ikari

**Affiliations:** 1 From the Laboratory of Biochemistry, Department of Biopharmaceutical Sciences, Gifu Pharmaceutical University, Gifu 501-1196, Japan; 116023@gifu-pu.ac.jp (H.S.); 125050@gifu-pu.ac.jp (A.T.); sendo@gifu-pu.ac.jp (S.E.); matsunagat@gifu-pu.ac.jp (T.M.); 2 Institute for Chemical Research, Kyoto University, Kyoto 611-0011, Japan; furuta@fos.kuicr.kyoto-u.ac.jp; 3 Nagaragawa Research Center, API Co., Ltd., Gifu 502-0071, Japan; ichihara-kenji@api3838.co.jp

**Keywords:** lung adenocarcinoma, claudin-2, kaempferol, chrysin, STAT3

## Abstract

Claudin-2 is highly expressed in human lung adenocarcinoma tissues and may be a novel target for cancer chemotherapy because knockdown of claudin-2 decreases cell proliferation. We found that flavonoids including kaempferol, chrysin, and luteolin concentration-dependently decrease claudin-2 expression in lung adenocarcinoma A549 cells. Claudin-2 expression is up-regulated by mitogen-activated protein kinase kinase (MEK)/ extracellular signal-regulated kinase (ERK)/c-Fos and phosphoinositide 3-kinase (PI3K)/Akt/nuclear factor-κB (NF-κB) pathways, but these activities were not inhibited by kaempferol, chrysin, and luteolin. Promoter deletion assay using luciferase reporter vector showed that kaempferol and luteolin inhibit the function of transcriptional factor that binds to the region between −395 and −144 of claudin-2 promoter. The decrease in promoter activity was suppressed by mutation in signal transducers and activators of transcription (STAT)-binding site, which is located between −395 and −144. The phosphorylation level of STAT3 was not decreased, but the binding of STAT3 on the promoter region is suppressed by kaempferol and luteolin in chromatin immunoprecipitation assay. The inhibition of cell proliferation caused by kaempferol and luteolin was partially recovered by ectopic claudin-2 expression. Taken together, kaempferol and luteolin decreased claudin-2 expression and proliferation in A549 cells mediated by the inhibition of binding of STAT3 on the promoter region of claudin-2. The intake of foods and nutrients rich in these flavonoids may prevent lung adenocarcinoma development.

## 1. Introduction

Epithelial cells form tight junctions (TJs) at the most apical pole of the lateral membrane between neighboring cells. The TJs regulate the flux of ions and solutes through the paracellular pathway, cell proliferation, polarization, and differentiation [1,2,3]. Most solid cancers are derived from epithelial tissues and disruption of polarity accelerates cell proliferation and differentiation. Claudins are integral membrane proteins of TJs and comprise a large family of 27 subtypes in a mammal [4,5]. Dysregulation of claudins expression has been shown in various tumor tissues [6]. We have been reported that the expression of claudin-2 is up-regulated in human lung adenocarcinoma tissues [7]. Similarly, the elevation of claudin-2 expression is reported in liver [8], colon [9], and stomach cancer tissues [10]. The knockdown of claudin-2 decreases proliferation and migration in non-small-cell lung carcinoma cells [7,11]. Therefore, claudin-2 may be one of the targets for anticancer function of foods and drugs.

Anticancer activity is reported in polyphenols extracted from plants, fruits, vegetables, and bee propolis [12,13]. Polyphenols include many classes of compounds ranging from flavonoids, phenolic acids, and anthocyanins. Some flavonoids inhibit growth and invasion of cancer cells in various in vitro and in vivo models. A population-based case-control study in Montreal, Canada, reported that a low dietary intake of flavonoids increases the risk of lung cancer [14]. Antioxidant effect is one of the key factors to reduce cancer risk [15]. Flavonoids have a basic structure consisting of two aromatic rings linked by three carbon atoms that are confined in an oxygenated heterocycle ring, and clarified as flavonols, flavones, flavanones, flavanols, anthocyanidins, and isoflavones [16]. The antioxidant activity is affected by the number and position of hydroxyl substituents [17]. Quercetin, with 3′,4′ dihydroxy substituents in the B ring and conjugation between the A and B rings has strong antioxidant activity, and shows apoptotic and anti-proliferative effects in human cancer cells derived from lung [18], breast [19], gastrointestinal tract [20], and prostate [21]. We previously reported that quercetin decreases claudin-2 expression in human lung adenocarcinoma A549 cells [22]. However, other flavonoids that decrease claudin-2 expression have not been identified.

The expression of claudins is controlled by various transcriptional factors under physiological and pathophysiological conditions. Signal transducers and activators of transcription (STAT) forms a family with seven members in mammals (STAT1, STAT2, STAT3, STAT4, STAT5A, STAT5B, and STAT6) [23]. Among them, STAT3 and STAT5 are activated in a large number of human cancers and play major roles in proliferation, apoptosis, and angiogenesis [24]. The activation of tyrosine kinase signal promotes dimerization of STAT3 and its translocation into the nuclei. STAT3 binds to the promoter regions of tumor-related genes [25]. A few reports indicate that STATs are involved in the regulation of claudins expression. The activation of STAT1 and STAT3 decreases claudin-5 expression in human brain microvascular endothelial cells [26] and claudin-2 expression in Madin-Darby canine kidney (MDCK) II cells [27], respectively, but the binding site of STAT on the promoter region of claudins is unknown.

In the present study, we searched for new substances that can decrease claudin-2 expression in A549 cells and identified kaempferol, chrysin, and luteolin as the potential candidates. To clarify the mechanism, we examined the effects of these flavonoids on intracellular signaling factors, promoter activity of claudin-2, binding of transcriptional factor on promoter of claudin-2, and cell proliferation. Our results indicate that kaempferol and luteolin may decrease claudin-2 expression through inhibiting the interaction of STAT3 on promoter region of claudin-2 without affecting the phosphorylation of STAT3, resulting in suppression of cell proliferation.

## 2. Experimental Section

### 2.1. Materials

Rabbit anti-claudin-1 and mouse anti-claudin-2 antibodies were obtained from Thermo Fisher Scientific (Waltham, MA, USA). Goat anti-β-actin and rabbit anti-phosphorylated-c-Fos (p-c-Fos) antibodies were from Santa Cruz Biotechnology (Santa Cruz, CA, USA). Rabbit anti-p-Akt, rabbit anti-Akt, rabbit anti-ERK1/2, rabbit anti-c-Fos, rabbit anti-p-NF-κB p65 (Ser536), and rabbit anti-NF-κB p65 antibodies were from Cell Signaling Technology (Beverly, MA, USA). Mouse anti-p-STAT3, anti-STAT3, and anti-nucleoporin p62 antibodies were from BD Biosciences (San Jose, CA, USA). Apigenin, genistein, and quercetin were from Wako Pure Chemical Industries (Osaka, Japan). Chrysin, kaempferol, daidzein, and 7-hydroxyflavone were from Tokyo Kasei Kogyo (Tokyo, Japan). Hesperetin was from LKT Laboratories (St. Paul, MN, USA). Luteolin and 4′-hydroxyflavone were from INDOFINE Chemical Company (Hillsborough, NJ, USA). Flavonoids were dissolved in dimethyl sulfoxide (DMSO). All other reagents were of the highest grade of purity available.

### 2.2. Cell Culture and Transfection

The human lung adenocarcinoma A549 cell line was obtained from the RIKEN BRC through the National Bio-Resource Project of the MEXT, Japan. The cells were grown in Dulbecco’s modified Eagle’s medium (DMEM, Sigma-Aldrich, St. Louis, MO, USA) supplemented with 5% fetal calf serum (FCS, HyClone, Logan, UT, USA), 0.07 mg/mL penicillin-G potassium, and 0.14 mg/mL streptomycin sulfate in a 5% CO_2_ atmosphere at 37 °C. The experiments were done in subconfluent culture condition (about 70–80% confluent), because the expression of claudin-2 decreased in 100% confluent condition [11]. The cells were treated with vehicle DMSO (control) and flavonoids (1–50 μM) for 24 h in FCS-free DMEM. The vector containing human claudin-2 cDNA was prepared as described previously [11]. The FLAG-tagged claudin-2 was sub-cloned into pTRE2-hyg vector and transfected into A549 cells with Lipofectamine 2000 as recommended by the manufacturer. Stable transfectants were selected with 500 ng/mL hygromycin B and maintained in the continuous presence of 50 ng/mL hygromycin B.

### 2.3. SDS-Polyacrylamide Gel Electrophoresis (SDS-PAGE) and Immunoblotting

Cells were scraped into cold phosphate-buffered saline and precipitated by centrifugation. They were lysed in a radioimmunoprecipitation assay lysis buffer containing 150 mM NaCl, 0.5 mM ethylenediaminetetraacetic acid, 1% Triton X-100, 0.1% sodium dodecyl sulfate, 50 mM Tris-HCl (pH 8.0), a protease inhibitor cocktail (Sigma-Aldrich), and sonicated for 20 s. The aliquots were used as whole cell extracts. After centrifugation at 6000× *g* for 5 min, the supernatants were collected and used as cell lysates which including plasma membrane and cytoplasmic proteins. Nuclear fractions were prepared using NE-PER nuclear and cytoplasmic fraction reagents as recommended by the manufacturer (Thermo Fisher Scientific). Samples were applied to SDS-PAGE and blotted onto a polyvinylidene difluoride membrane. The membrane was then incubated with each primary antibody (1:1000 dilution) at 4 °C for 16 h, followed by a peroxidase-conjugated secondary antibody (1:5000 dilution) at room temperature for 1 h. Finally, the blots were incubated in Pierce Western Blotting Substrate (Thermo Fisher Scientific) and exposed to film, or incubated in ECL Prime Western Blotting Detection System (GE healthcare, Chalfont St Giles, UK) and scanned with a C-DiGit Blot Scanner (LI-COR Biotechnology, Lincoln, NE, USA). Blots were further stripped and reprobed with anti-β-actin antibody. Band density was quantified with ImageJ software (National Institute of Health software, NIH, Bethesda, MD, USA). The signals were normalized for the loading control β-actin or nucleoporin p62. The expression levels were represented relative to the values in the absence of flavonoids.

### 2.4. Measurement of O_2_^−^ Scavenging Activity

Antioxidant activity of flavonoids and antioxidants was measured using the hypoxanthine-xanthine oxidase system as the source of superoxide anion [28]. Reaction solution contains 10 μM 2-methyl-6-p-methoxyphenyl ethynylimidazopyrazynone, 0.02 units/mL xanthine oxidase, 0.12 mM hypoxanthine, and 20 mM KH_2_PO_4_ (pH 7.5). Test compounds were mixed in the reaction buffer at the final concentration of 50 μM. A chemiluminescence intensity was measured with a luminometer (AB-2270 Luminescencer Octa, ATTO, Tokyo, Japan). O_2_^−^ scavenging activity was calculated by the following formula: Scavenging activity (%) = (1 − CL_S_/CL_C_) × 100; where CL_C_, chemiluminescence of control, CL_A_, chemiluminescence of sample.

### 2.5. RNA Isolation and Polymerase Chain Reaction (PCR)

Total RNA was isolated from A549 cells using TRI reagent (Sigma-Aldrich). Reverse transcription was carried out with ReverTra Ace qPCR RT Kit (Toyobo Life Science, Osaka, Japan). Semi-quantitative PCR was carried out with DNA Engine Dyad Cycler (Bio-Rad, Richmond, CA, USA) using GoTaq DNA polymerase (Promega, Madison, WI, USA). The PCR product was visualized with ethidium bromide after electrophoretic separation on a 2% agarose gel. The size of PCR product was 86 bp (claudin-2) and 100 bp (β-actin). Quantitative real-time PCR was performed with a Thermal Cycler Dice Real-time System (TP700, Takara Bio, Shiga, Japan) or Eco Real-Time PCR system (AS One, Osaka, Japan) using KOD SYBR qPCR Mix (Toyobo Life Science). The primers used to PCR are listed in Table 1. The threshold cycle (Ct) for each PCR product was calculated with the instrument’s software, and Ct values obtained for claudin-1 and -2 were normalized by subtracting the Ct values obtained for β-actin. The resulting ∆Ct values were then used to calculate the relative change in mRNA expression as a ratio (R) according to the equation R = 2^−(∆Ct(treatment)^^−∆Ct(control))^.

### 2.6. Luciferase Reporter Assay

Using the reporter plasmids containing fragments of −1031/+37, −393/+37, −143/+37, and −87/+37 of human claudin-2, luciferase reporter assay was carried out as described previously [29]. The mutant of putative STAT-binding site (−252/−230) was generated using the primer pairs (forward: 5′-GAATCTCGAGCAGCCACCTGTCTGGCTCCTGGC-3′ and reverse: 5′-AGGTGATGATGGCAGTGGTGGTTGTG-3′) and KOD-Plus-mutagenesis kit (Toyobo Life Science). The luminescence of luciferase was measured with a luminometer.

### 2.7. Chromatin Immunoprecipitation (ChIP) Assay

Cells were treated with 1% formaldehyde to crosslink the protein to the DNA. ChIP assay was carried out as described previously [29]. To co-immunoprecipitate the DNA, anti-STAT3 antibody or mouse IgG was used. The eluted DNA was amplified by quantitative PCR using the primer pairs for claudin-2 promoter (forward: 5′-ACTTGAGTTAACACAGCCACCA-3′ and reverse: 5′-ACTTTGAACGTGGAGCCAAAAT-3′). To confirm the same amounts of chromatins used in immunoprecipitation between groups, input chromatin was also used.

### 2.8. Cell Proliferation

Cells were seeded at 1 × 10^5^ cells in 60-mm dishes. After 24, 48, and 72 h of culture, the cell images were captured with a light microscope (CKX53, Olympus, Tokyo, Japan). Cell proliferation was calculated by counting the number of cells using the CKX-CCSW software (Olympus).

### 2.9. Statistics

Results are presented as means ± S.E.M. Differences between groups were analyzed with a one-way analysis of variance, and corrections for multiple comparison were made using Tukey’s multiple comparison test. Comparisons between two groups were made using Student’s *t*-test. Statics were performed using KaleidaGraph version 4.5.1 software (Synergy Software, Reading, PA, USA). Significant differences were assumed at *p* < 0.05.

## 3. Results

### 3.1. Effects of Flavonoids on Claudin-2 Expression in A549 Cells

**Figure 1:**
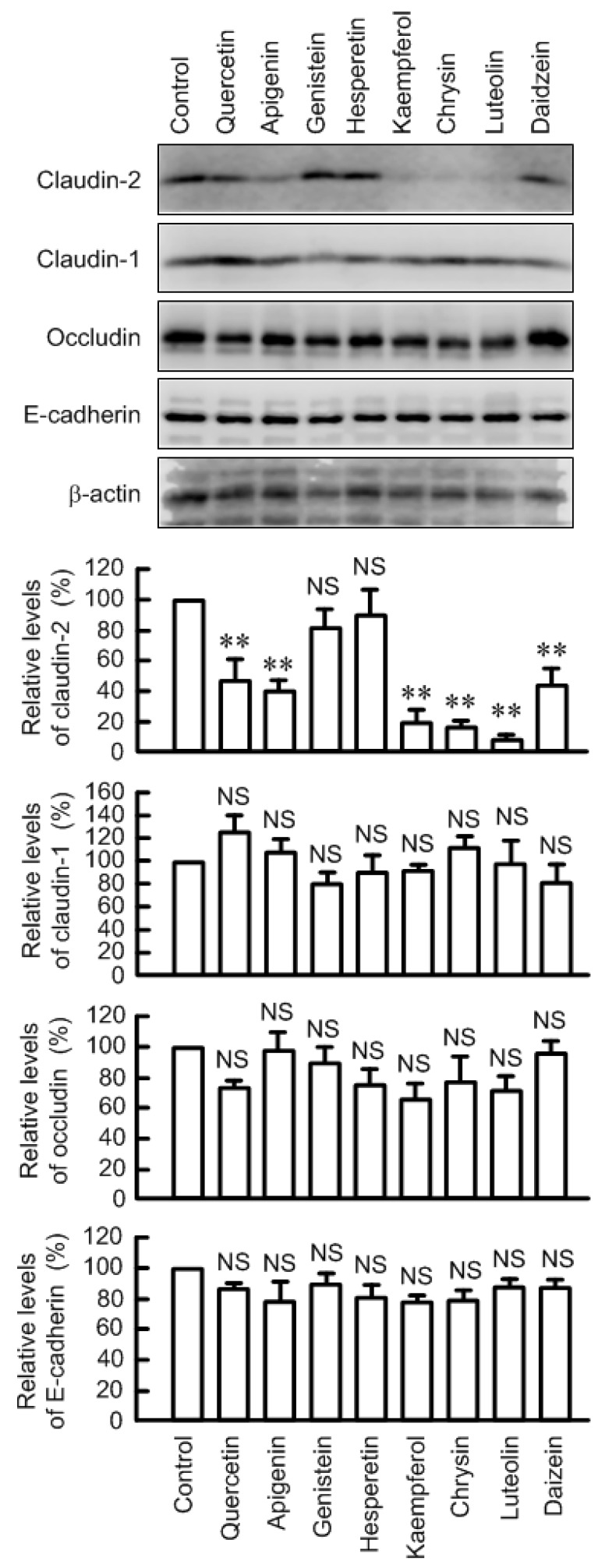
Effects of flavonoids on expression of junctional proteins in A549 cells. Cells were incubated in the absence and presence of 50 μM flavonoids including quercetin, apigenin, genistein, hesperetin, kaempferol, chrysin, luteolin, and daidzein for 24 h. Cell lysates were immunoblotted with anti-claudin-2, anti-claudin-1, anti-occludin, anti-E-cadherin, or anti-β-actin antibody. The expression levels were represented relative to the values in control. *n* = 3–4. ** *p* < 0.01 significantly different from control. NS, not significantly different.

The protein level of claudin-2 in the cytoplasmic fraction was significantly decreased by quercetin, apigenin, kaempferol, chrysin, luteolin, and daizein at the concentration of 50 μM in A549 cells (Figure 1). The effects of kaempferol, chrysin, and luteolin were stronger than those of other flavonoids. Genistein and hesperetin showed no effect on claudin-2 expression. Hesperetin, kaempferol, and luteolin showed cytotoxicity at over 100 μM, but all flavonoids have little effect on cytotoxicity at the concentration of lower than 50 μM (Supplementary Figure S1). These results indicate that the decrease in claudin-2 expression is not due to cytotoxicity. The expression of other junctional proteins including claudin-1, occludin, and E-cadherin was not changed by these flavonoids. In the present study, we focused on the regulatory mechanism of which kaempferol, chrysin, and luteolin decrease claudin-2 expression because the effects of these flavonoids are the strongest.

### 3.2. Effects of Antioxidant Capacity on Claudin-2 Expression

**Figure 2:**
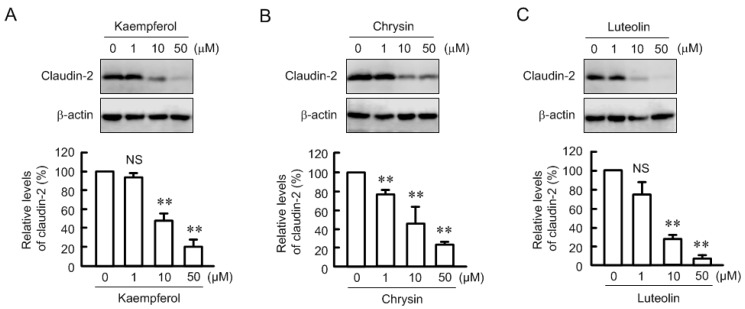
Dose-dependent effects of kaempferol, chrysin, and luteolin on claudin-2 expression. The cells were incubated in the absence and presence of kaempferol (**A**), chrysin (**B**), or luteolin (**C**) for 24 h at the concentration indicated. Cell lysates were immunoblotted with anti-claudin-2 or β-actin antibody. The expression levels of claudin-2 were represented relative to the values in 0 μM. *n* = 3–4. ** *p* < 0.01 significantly different from 0 μM. NS, not significantly different.

**Figure 3:**
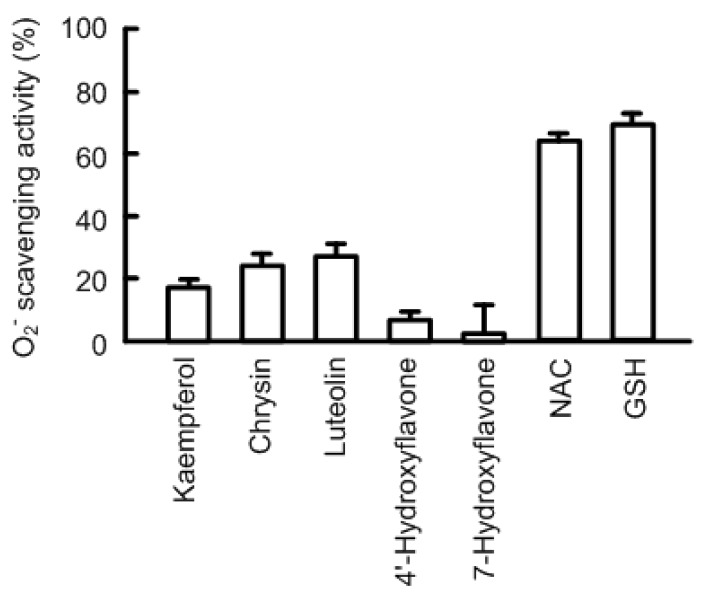
Antioxidant effects of flavonoids and antioxidants. O_2_^−^ scavenging activity was examined using the hypoxanthine-xanthine oxidase system. Flavonoids (50 μM), *N*-acetyl cysteine (NAC, 1 mM), and reduced glutathione (GSH, 0.1 mM) were mixed in the reaction buffer. *n* = 4.

**Figure 4:**
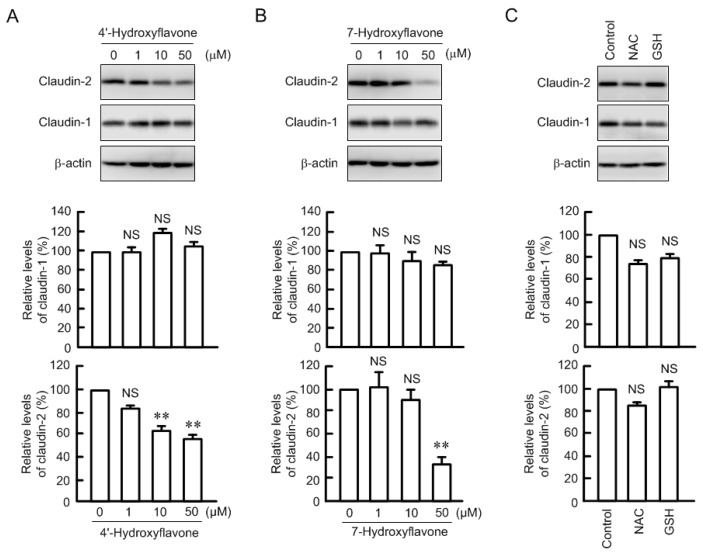
Effects of non-antioxidative flavones and antioxidants on claudin-2 expression. (**A**,**B**) The cells were incubated in the absence and presence of 4′-hydroxyflavone or 7-hydroxyflavone for 24 h at the concentration indicated. Cell lysates were immunoblotted with anti-claudin-1, claudin-2, or β-actin antibody. The expression levels of claudin-1 and -2 were represented relative to the values in 0 Μm; (**C**) Cells were incubated in the absence (control) and presence of NAC (1 mM) or GSH (0.1 mM) for 24 h. Cell lysates were immunoblotted with anti-claudin-1, claudin-2, or β-actin antibody. The expression levels of claudin-1 and -2 were represented relative to the values in control. *n* = 3. ** *p* < 0.01 significantly different from 0 μM or control. NS, not significantly different.

The expression of claudin-2 was dose-dependently decreased by kaempferol, chrysin, and luteolin (Figure 2). The effects were significant over the concentration of 1 or 10 μM. Certain flavonoids have an antioxidant effect. Kaempferol, chrysin, and luteolin showed approximately 20% O_2_^−^ scavenging activity at 50 μM (Figure 3). 4′-Hydroxyflavonoe and 7-hydroxyflavone had little antioxidant activity, which is similar to previous report [30]. In contrast, *N*-acetyl cysteine (NAC, 1 mM) and reduced glutathione (GSH, 0.1 mM) had strong antioxidant activity. Although 4′-hydroxyflavonoe and 7-hydroxyflavone had little antioxidant activity, they decreased claudin-2 expression (Figure 4). In addition, claudin-2 expression was not decreased by NAC and GSH. These results indicate that antioxidant capacity may not be involved in the decrease in claudin-2 expression by flavonoids.

### 3.3. Effects of Flavonoids on mRNA Levels of Junctional Protein and Intracellular Signaling Pathway

**Figure 5:**
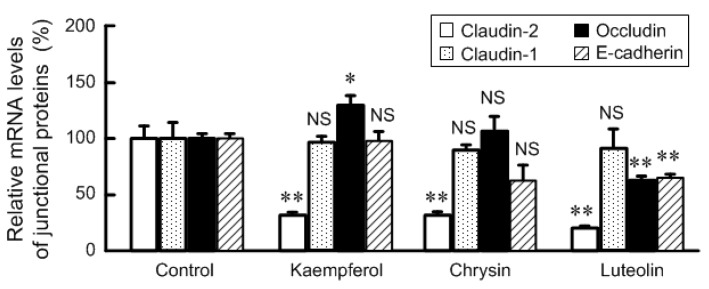
Effects of flavonoids on mRNA levels of junctional protein. The cells were incubated in the absence and presence of 50 μM flavonoids for 6 h. After isolation of total RNA, quantitative real-time PCR was performed using specific primers for claudin-1, claudin-2, occludin, and E-cadherin. *n* = 4. ** *p* < 0.01 and * *p* < 0.05 significantly different from control. NS, not significantly different.

**Figure 6:**
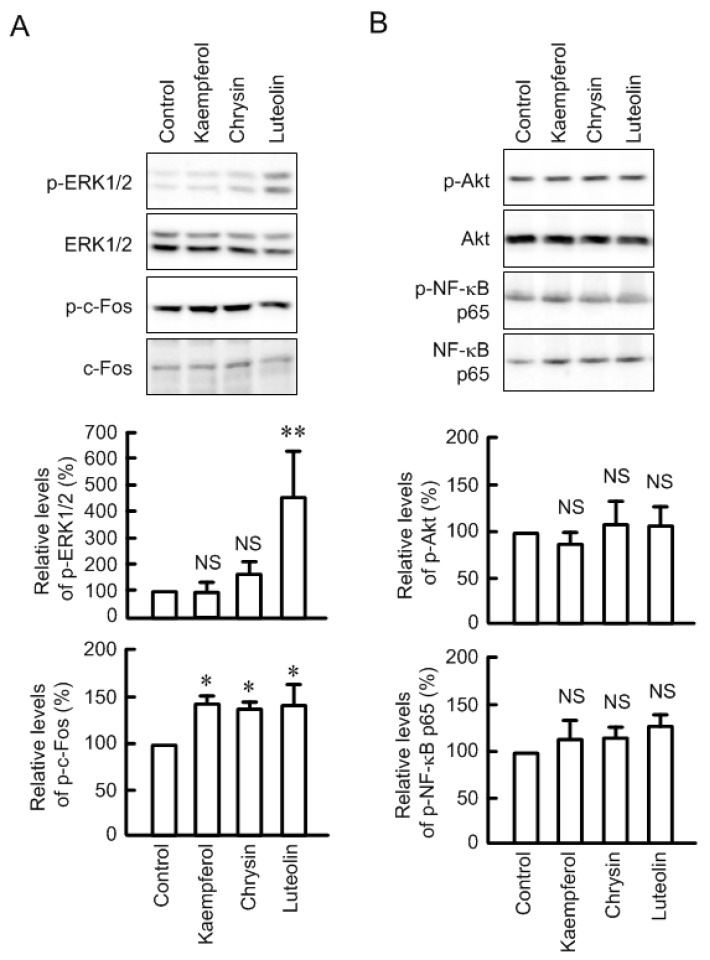
Effects of flavonoids on the phosphorylation of intracellular signaling pathways. The cells were incubated in the absence and presence of 50 μM flavonoids for 1 h. (**A**) Whole cell extracts were immunoblotted with anti-p-ERK1/2, ERK1/2, p-c-Fos, or c-Fos antibody; (**B**) Whole cell extracts were immunoblotted with anti-p-Akt, Akt, p-NF-κB p65, or NF-κB p65 antibody. The levels of p-ERK, p-c-Fos, p-Akt, and p-NF-κB p65 were represented relative to the values in control. *n* = 3. ** *p* < 0.01 and * *p* < 0.05 significantly different from control. NS, not significantly different.

Kaempferol, chrysin, and luteolin significantly decreased the mRNA level of claudin-2 without changing that of claudin-1 (Figure 5). These results coincide with those of Western blotting. In contrast, the mRNA level of occludin was increased by kaempferol and those of occludin and E-cadherin were decreased by luteolin. We previously reported that the transcriptional activity of claudin-2 is up-regulated by the MEK/ERK/c-Fos [29] and PI3K/Akt/NF-κB pathways [31]. Some flavonoids, including kaempferol, chrysin, and luteolin, increased the phosphorylation levels of ERK1/2 and/or c-Fos (Figure 6). On the contrary, these flavonoids had no effect on the phosphorylation of Akt and NF-κB p65. These results indicate that the MEK/ERK/c-Fos and PI3K/Akt/NF-κB pathways may not be involved in these flavonoids-induced decreases in claudin-2 expression.

### 3.4. Effects of Flavonoids on the Promoter Activity of Claudin-2

**Figure 7:**
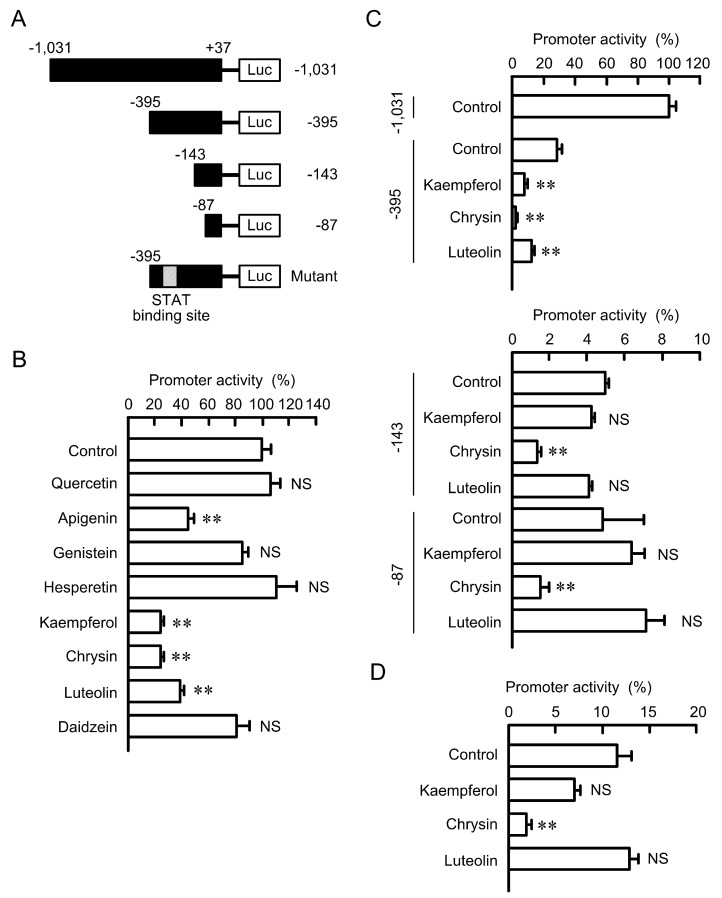
Effects of flavonoids on the promoter activity of human claudin-2. (**A**) Schematic drawing of human claudin-2 luciferase reporter vector; (**B**) The cells were co-transfected with luciferase −1031/pGL4.10 and pRL-TK vectors. After 24 h of transfection, the cells were incubated in the absence and presence of 50 μM flavonoids for 24 h. The promoter activity of claudin-2 was represented relative to the values in control; (**C**) The constructs of 5′-deletion series including −395, −143, and −87/pGL4 vector were co-transfected with the pRL-TK vector; (**D**) The construct of mutant of STAT-binding site was co-transfected with the pRL-TK vector. The relative promoter activity was represented relative to the values of control of −1031/pGL4 vector. *n* = 3–4. ** *p* < 0.01 significantly different from vehicle. NS, not significantly different.

To clarify a major transcriptional factor involved in the flavonoids-induced decreases in claudin-2 expression, we performed the promoter deletion assay of human claudin-2. Apigenin, kaempferol, chrysin, and luteolin significantly decreased the promoter activity (Figure 7). These results coincide with those of Western blotting. In contrast, quercetin, genistein, hesperetin, and daidzein did not inhibit the promoter activity. Kaempferol, chrysin, and luteolin inhibited the activity of construct of −395/+37, but the inhibitory effects of kaempferol and luteolin disappeared in the constructs of −143/+37 and −87/+37. In contrast, the promoter activity of all deletion constructs was significantly inhibited by chrysin. These results indicate that the kaempferol- and luteolin-sensitive transcriptional factor may bind to the region between −395 and −144, and chrysin-sensitive one may bind to the region between −86 and −1. TFSEARCH showed that the region between −395 and −144 of claudin-2 promoter contains a putative STAT-binding site. The promoter activity of mutant of STAT-biding site was not significantly inhibited by kaempferol and luteolin. These results indicate that STAT may be involved in the decrease in claudin-2 expression by kaempferol and luteolin.

### 3.5. Inhibition of Association between STAT3 and Claudin-2 Promoter by Flavonoids

**Figure 8:**
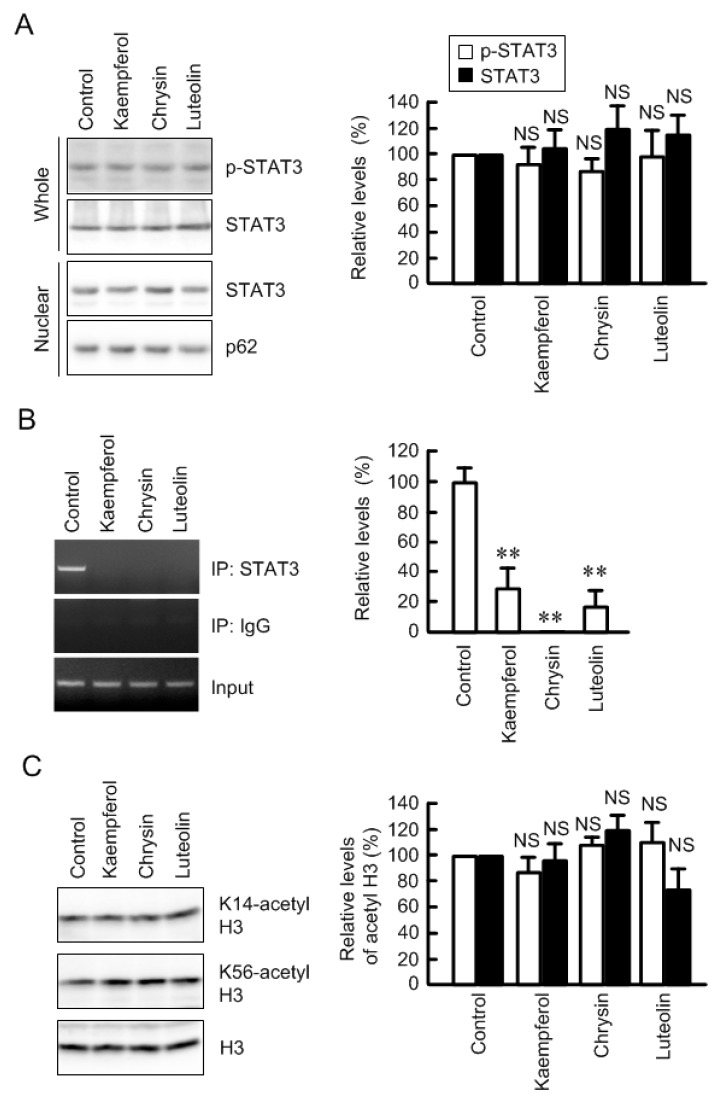
Effects of flavonoids on the binding of STAT3 to promoter region of claudin-2. The cells were incubated in the absence (control) and presence of 50 μM flavonoids for 1 h. (**A**) Whole cell extracts and nuclear fractions were immunoblotted with anti-p-STAT3, anti-STAT3, or anti-nucleoporin p62 antibody. The levels of p-STAT3 were represented relative to the values in control; (**B**) After immunoprecipitation of genomic DNA by anti-STAT3 antibody, semi-quantitative PCR (left images) and quantitative real-time PCR (right graphs) were performed using the primers amplifying the putative STAT-binding site of claudin-2 promoter. Mouse IgG was used for negative control. Input chromatin was used for normalization. The amount of PCR products is represented relative to the value of control cells; (**C**) Nuclear fractions were immunoblotted with anti-acetyl histone H3 (K14 or K56) or anti-histone H3 antibody. The levels of acetyl histone H3 were represented relative to the values in control. *n* = 3. ** *p* < 0.01 significantly different from control. NS, not significantly different.

STAT3 is abundantly expressed in lung cancer cells and involved in the development of cancer [32]. The phosphorylation and nuclear localization of STAT3 were not inhibited by kaempferol, chrysin, and luteolin (Figure 8). In the ChIP assay, a primer pairs amplifying the STAT-binding site showed positive PCR signals in the control cells using anti-STAT3 antibody (Figure 8B). In the kaempferol, chrysin, and luteolin-treated cells, PCR signal was faint. The primer pairs amplifying STAT-binding site showed PCR bands using input samples. These results indicate that these flavonoids inhibit interaction between STAT3 and claudin-2 promoter. The interaction between transcriptional factor and promoter region is often regulated by epigenetic modification, such as histone acetylation [33]. However, three flavonoids did not change acetylation levels of histone H3 (Figure 8C), indicating that epigenetic mechanism may not be involved in the inhibition of association between STAT3 and claudin-2 promoter.

### 3.6. Effects of Three Flavonoids and Ectopic Claudin-2 Expression on Cell Proliferation

**Figure 9:**
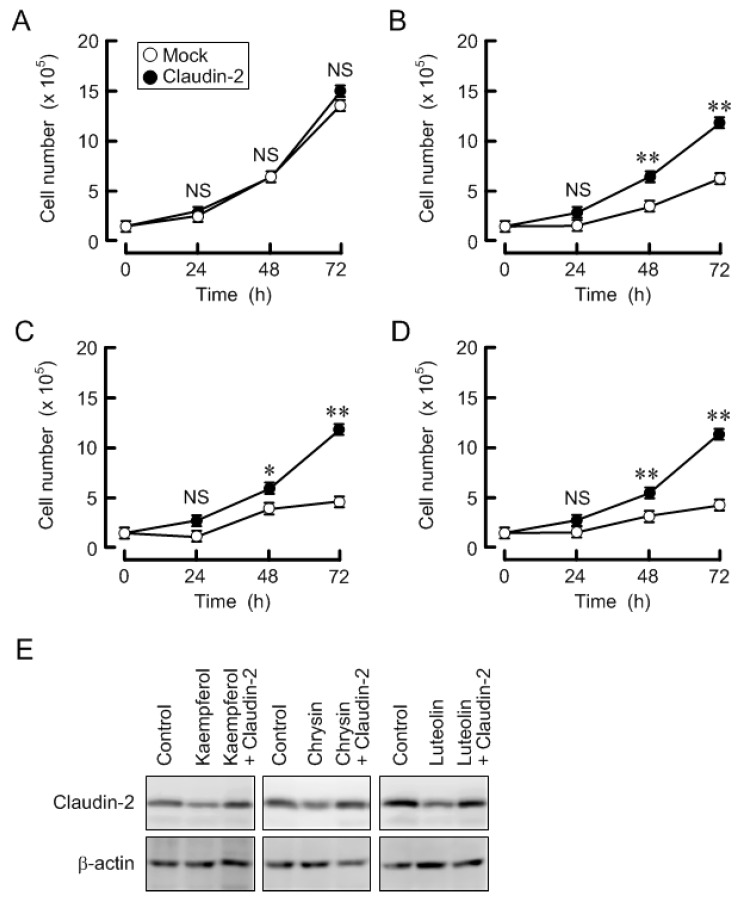
Rescue of cell proliferation by ectopic claudin-2 expression. Mock and claudin-2-overexpressing cells were cultured in the absence (**A**) and presence of 10 μM kaempferol (**B**); chrysin (**C**); or luteolin (**D**). Cell number was measured at 24, 48, and 72 h after inoculation; (**E**) Cell lysates were immunoblotted with anti-claudin-2 or β-actin antibody. *n* = 3–4. ** *p* < 0.01 and * *p* < 0.05 significantly different from mock. NS, not significantly different.

Claudin-2 is partially involved in the regulation of proliferation in A549 cells [7,11]. Kaempferol, chrysin, and luteolin decreased claudin-2 expression, so we examined the effects of these flavonoids on cell proliferation. To avoid the effect of cytotoxicity, the cells were treated with these flavonoids at the concentration of 10 μM (Supplementary Figure S1). Cell number was not changed by overexpression of claudin-2 in the absence of flavonoids (Figure 9). Three flavonoids inhibited the increase in cell number and decreased the expression of claudin-2, which were rescued by ectopic claudin-2 expression. Therefore, these results indicate that kaempferol, chrysin, and luteolin may inhibit cell proliferation mediated by the decrease in claudin-2 expression.

## 4. Discussion

The effects of flavonoids on the barrier function and expression of claudins have been examined in epithelial cells. Quercetin enhances intestinal barrier in Caco-2 cells through the elevation of claudin-4 [34] and through the assembly of zonula occludens-2, occludin, and claudin-1 [35]. Genistein and daidzein protect lipopolysaccharide-induced disruption of barrier function in glandular endometrial epithelial cells through the elevation of claudin-3, -4, and -8 [36]. In contrast, the effects of flavonoids on the pathophysiological function and expression of claudins in cancer cells have not been examined well. Here, we found that kaempferol, chrysin, and luteolin decrease the mRNA and protein levels of claudin-2 in lung adenocarcinoma A549 cells. The expression of claudin-2 is decreased by chemical inhibitors and dominant negative plasmids of MEK/ERK/c-Fos and PI3K/Akt/NF-κB pathways in A549 cells [7,31]. These factors of intracellular signaling pathway are activated in the lung cancer tissues [37,38]. However, kaempferol, chrysin, and luteolin did not inhibit the phosphorylation of ERK1/2 and Akt, but they surprisingly increased these phosphorylation. We suggest that kaempferol, chrysin, and luteolin decrease claudin-2 expression mediated by the different mechanisms from MEK/ERK/c-Fos and PI3K/Akt/NF-κB pathways.

The regulatory mechanism of claudin-2 expression is different in each tissue. Claudin-2 expression is down-regulated by the activation of MEK/ERK pathway in renal tubular Madin Darby canine kidney type II (MDCK II) cells [39]. The effect of MEK/ERK pathway in MDCK II cells is opposite to that in A549 cells [29]. At present, it is unknown why the MEK/ERK pathway has contrary effects on claudin-2 expression in both cells. In intestinal Caco-2 cells, claudin-2 is up-regulated by caudal-related homeobox 2 (cdx2) [40]. Cdx2 is an intestine-specific transcription factor highly expressed in the tissues of dysplasia and cancer, but is not expressed in the lung. Therefore, cdx2 should not be involved in the up-regulation of claudin-2 in lung cancer. To identify novel compounds that decrease claudin-2 expression, we had to search for them based on the novel mechanism of action.

It has been postulated that oxidative stress is closely associated with tumor formation, progression and metastasis. Numerous naturally occurring anti-oxidant compounds possess anti-cancer properties [12]. Oxidative stresses including hydrogen peroxide and nitric oxide decrease claudin-1 and -5 expression in colonic HT-29 cells [41], claudin-2 expression in MDCK II cells [42], and claudin-5 expression in ischemic brain [43], which are inhibited by antioxidants. Oxidative stress may be one of the key factors controlling claudins expression. Kaempferol, chrysin, and luteolin showed little antioxidative effect at the concentration used in the present study (Figure 3), whereas NAC and GSH had strong antioxidant activity. Nevertheless, both NAC and GSH did not decrease claudin-2 expression, whereas 4′-hydroxyflavonoe and 7-hydroxyflavone dose-dependently decreased. These results suggest that anti-oxidative capacity is not necessary to decrease claudin-2 expression in A549 cells, but the basic structure of flavones or flavonols is necessary.

Kaempferol, chrysin, and luteolin have been reported to inhibit properties of cancer mediated by inhibition of phosphorylation of STAT3: Kaempferol induces differentiation in partially differentiated colon cancer cells [44], chrysin suppresses hypoxia-induced metastasis of 4T1 mouse breast cancer cells [45], and luteolin inhibits hypoxia-induced vascular endothelial factor expression [46]. In contrast, our results indicated that these flavonoids do not inhibit the phosphorylation of STAT3, but they block the interaction between STAT3 and the promoter region of claudin-2. The direct inhibition of the binding of transcriptional factor and DNA are reported by some flavonoids. Apigenin, chrysin, and kaempferol bind to peroxisome proliferator-activated receptor γ and suppress inducible cyclooxygenase (COX) and nitric oxide synthase promoter activity in mouse macrophages [47]. Chrysin binds to nuclear factor for IL-6 and suppresses COX-2 expression in lipopolysaccharide-activated Raw 264.7 cells [48]. The binding site of flavonoids on STAT3 is unknown, but kaempferol, chrysin, and luteolin could interfere the interaction between STAT3 and promoter region of claudin-2.

The pharmacological inhibitor and dominant-negative isoform of STAT3 have been reported to induce cell cycle arrest in A549 cells [32]. So far, we reported that knockdown of claudin-2 inhibits cell cycle G1/S transition without affecting cytotoxicity [11]. In the present study, kaempferol, chrysin, and luteolin showed little cytotoxicity (Supplementary Figure S1) and decreased cell number. The flavonoids-induced decrease in cell number was significantly rescued by ectopic claudin-2 expression. Therefore, we suggest that these flavonoids inhibit cell proliferation mediated by the reduction of claudin-2 expression.

## 5. Conclusions

In the present study, we found that kaempferol, chrysin, and luteolin decrease claudin-2 expression in A549 cells. Kaempferol and luteolin inhibited the promoter activity of human claudin-2, which was rescued by deletion or mutation of STAT-binding site. The phosphorylation and nuclear localization of STAT3 were not inhibited by kaempferol and luteolin, but the interaction between STAT3 and the promoter region of claudin-2 was inhibited, indicating that kaempferol and luteolin may directly block the interaction of STAT3 on DNA. Chrysin also inhibited the promoter activity of claudin-2, which was not rescued by deletion of STAT-binding site. Other important transcriptional factors, which bind to the region between −86 and −1 of claudin-2 promoter, should be involved in the inhibitory effect of chrysin. Some foods that abundantly include these flavonoids may be useful to prevent development of lung adenocarcinoma.

## Figures and Tables

**Table 1:** Primers for polymerase chain reaction (PCR) amplification.

Name	Direction	Sequence
Claudin-1	Forward	5′-ATGAGGATGGCTGTCATTGG-3′
Claudin-1	Reverse	5′-ATTGACTGGGGTCATAGGGT-3′
Claudin-2	Forward	5′-ATTGTGACAGCAGTTGGCTT-3′
Claudin-2	Reverse	5′-CTATAGATGTCACACTGGGTGATG-3′
Occludin	Forward	5′-TTTGTGGGACAAGGAACACA-3′
Occludin	Reverse	5′-TCATTCACTTTGCCATTGGA-3′
E-cadherin	Forward	5′-ACCCCCTGTTGGTGTCTTT-3′
E-cadherin	Reverse	5′-TTCGGGCTTGTTGTCATTCT-3′
β-actin	Forward	5′-CCTGAGGCACTCTTCCAGCCTT-3′
β-actin	Reverse	5′-TGCGGATGTCCACGTCACACTTC-3′

## Supplementary Materials

The following are available online at www.mdpi.com/2072-6643/9/6/597/s1, Figure S1: Effects of flavonoids on cell viability.

## Abbreviations

cdx2	caudal-related homeobox 2
ChIP	chromatin immunoprecipitation
COX	cyclooxygenase
DMEM	Dulbecco’s Modified Eagle’s medium
DMSO	Dimethyl sulfoxide
ERK	extracellular signal-regulated kinase
FCS	fetal calf serum
NAC	*N*-acetyl cysteine
MDCK	Madin Darby canine kidney
MEK	mitogen-activated protein kinase kinase
NF-κB	nuclear factor-κB
PCR	polymerase chain reaction
PI3K	phosphoinositide 3-kinase
SDS-PAGE	SDS-polyacrylamide gel electrophoresis
STAT	signal transducers and activators of transcription
TJs	tight junctions
