# Supplementary material for: Kaempherol and Luteolin Decrease Claudin-2 Expression Mediated by Inhibition of STAT3 in Lung Adenocarcinoma A549 Cells
Source: Nutrients. 2017 Jun 13;9(6):597. doi: 10.3390/nu9060597 (PMC5490576; doi:10.3390/nu9060597)
Supplement: Supplementary file 1 [file nutrients-09-00597-s001.zip › nutrients-192407-supplementary.pdf]

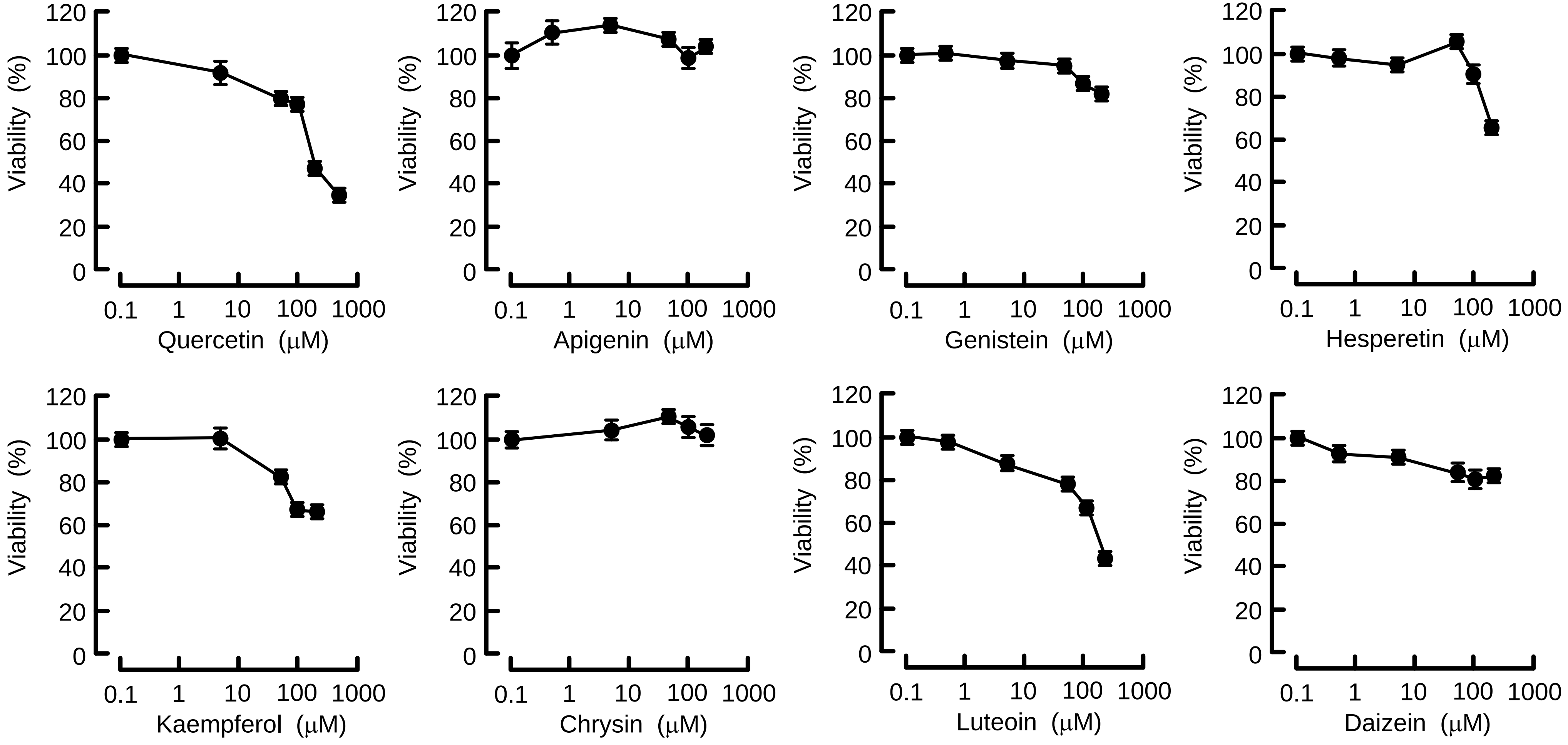

Supplementary figure 1. Effects of flavonoids on cell viability. A549 cells were incubated in the absence and presence of 50 μM flavonoids for 24 h at the concentration indicated. Cell viability was assessed by WST-1 assay.
